# Retrospective Multivariate Analysis of Data from Children with Suspected Appendicitis: A New Tool for Diagnosis

**DOI:** 10.1155/2018/4810730

**Published:** 2018-09-12

**Authors:** Zafer Dokumcu, Bade Toker Kurtmen, Emre Divarci, Petek Bayindir Tamay, Timur Kose, Murat Sezak, Geylani Ozok, Orkan Ergun, Ahmet Celik

**Affiliations:** ^1^Ege University Faculty of Medicine Department of Pediatric Surgery, Izmir, Turkey; ^2^Ege University Faculty of Medicine Department of Radiology, Division of Pediatric Radiology, Izmir, Turkey; ^3^Ege University Faculty of Medicine Department of Biostatistics and Medical Informatics, Izmir, Turkey; ^4^Ege University Faculty of Medicine Department of Pathology, Izmir, Turkey

## Abstract

**Background:**

Decision-making for management may sometimes be difficult in acute appendicitis (AA). Various diagnostic scoring systems exist, but their sensitivity and specificity rates are far from ideal. In this study, the determination of the predictors and the effect of radiological data and developing a new scoring system were aimed.

**Methods:**

Medical records of patients who were hospitalized for AA between February 2012 and October 2016 were retrospectively reviewed. All data were compared between patients with and without appendicitis. The multivariate analysis was performed to define significant variables and to examine the sensitivity and specificity of each group of predictors including radiological data. A new scoring system (NSS) was formed and was compared with two existing scoring systems: pediatric appendicitis score (PAS) and Alvarado scoring system (ASS) by using reclassification method.

**Results:**

Negative appendectomy rate was 11.3%. Statistical analysis identified 21 independently significant variables. The heel drop test had the highest odds ratio. Sensitivity and specificity rates of clinical predictors were 84.6% and 94.8%, respectively. Radiological predictors increased the sensitivity rate to 86.9%. Sensitivity and specificity rates for PAS, ASS, and NSS were 86.8% and 83.9%, 84.7% and 81.6%, and 96.8% and 95.6%, respectively. The “re-assessed negative appendectomy rate” was 6.2% and false positive results were remarkably more common in patients with duration of symptoms less than 24 hours.

**Conclusion:**

Radiological data improves the accuracy of diagnosis. Containing detailed clinical and radiological data, NSS performs superiorly to PAS and ASS, regarding sensitivity and specificity without any age limitation. The efficiency of NSS may be enhanced by determining different predictors for different phases of the inflammatory process.

## 1. Introduction

Acute appendicitis (AA) is the most common abdominal surgical emergency in children. Due to its progressive inflammatory pathophysiological course and anatomical variations of the appendix, it may occur in various clinical forms. The decision for surgical exploration is often made upon the clinical course, whereas laboratory and radiological tests are useful in most cases [1–4]. Routine laboratory tests include hemogram, spot urine test, plain X-ray, and ultrasonography. Computerized tomography may also be used, but its disadvantages of exposure to radiation and high cost make its use debatable [4, 5].

Pediatric appendicitis score (PAS) and Alvarado scoring system (ASS) were commonly cited for a standardized approach in children with suspected AA [6, 7]. These systems are mainly based on a limited number of symptoms and signs on physical examination and white blood count, excluding radiological data. Their use remained relatively limited to emergency departments to distinguish between patients to be consulted by the surgical team due to much less sensitivity and specificity ratios in referring studies than the original articles [8]. Besides, there are recent studies to suggest that radiological information additive to clinical evaluation increases the chance for right decision-making in these children [9, 10].

In the era of evidence-based medicine, the clinician should benefit from every available data before the decision of surgical exploration for AA while consideration for legal-ethical issues and cost-effectiveness remains critical. The hypothesis of this preliminary study is a standardized approach including routine radiological assessment of patients with suspicion of appendicitis increases the success rate. The assessment of the reliability of each symptom, findings on physical examination and radiological data, and evaluating the efficiency of radiological input in the diagnosis of AA are the primary objectives. Secondary objective is the formation of a new scoring system with superior sensitivity and specificity than existing scoring systems.

## 2. Material and Methods

After approval of the institutional review board (IRB#18-2.1/24), medical records of patients with suspected AA were retrospectively reviewed. The cross-sectional study was conducted over four years (February 2012–October 2016) at a large urban tertiary center with five board-certified surgeons. All children (1-18 years old) who were admitted with clinically suspected AA were included. Children with incomplete medical records and patients with pathologies of other than AA were excluded.

**Figure 1:**
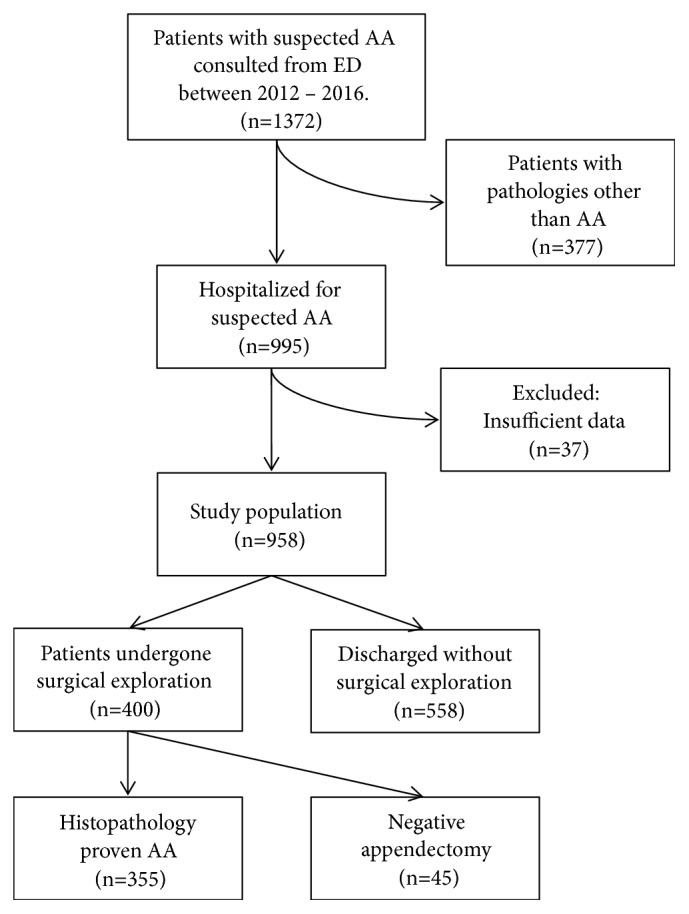
Flowchart of patients included in the study (AA: acute appendicitis, ED: Emergency Department).

All suspected AA patients were hospitalized for either urgent surgical exploration or clinical observation. A detailed history of symptoms, a thorough physical examination, routine laboratory tests, and imaging modalities (abdominal radiography and ultrasonography) were obtained and recorded on prestructured evaluation forms for all patients.  Figure 1 shows the flowchart of the study population.

Gender, type (continuous or intermittent), duration and migration of abdominal pain, nausea, bilious vomiting, changes in defecation, pyrexia, urinary and bowel habit changes, and menstrual status for girls were questioned in the history. Localized abdominal tenderness, pain on percussion and guarding, gurgling, positive heel drop test, and alteration of bowel movements were noted. Leucocytosis (>10.500/mm^3^) and neutrophilia (>75%), elevated levels of C-reactive protein (CRP), and leukocyturia in urinalysis were checked. Scoliosis to the right side, localized air-fluid level or gas deposition on the right lower quadrant, and fecalith on standing abdominal X-ray were noted. Ultrasonographic appendix diameter (>7mm), presence of thickened wall, and surrounding loculated fluid collection were evaluated.

The decision for surgery was made upon clinical and radiological evaluations or repeated physical examinations. Appendectomies were performed either by a board-certified surgeon or by a resident under the supervision of a board-certified surgeon. The modality of surgical exploration (open surgery or laparoscopy) differed according to the surgeon's preference. The existence of polymorphonuclear leukocytes and lymphocytes in the appendiceal specimen was considered positive for AA. Negative appendectomy was defined as the absence of inflammatory cells in the appendiceal sample.

Patients were grouped into two groups: Group Appendicitis (Group A) and Group Nonappendicitis (Group NA). Group A included patients who were operated, and diagnosis of AA was confirmed by the histopathological evaluation. Group NA included patients that were discharged without operation after repeated physical examinations and patients with negative appendectomy (appendix vermiformis).

All prestructured forms were collected, and data were transferred to Excel 2010 (Microsoft, Redmond WA, USA) format. Continuous variables were presented as mean ± standard deviation and data were compared using an unpaired t-test. Categorical variables were expressed as numbers and percentages and analyzed for comparisons using Pearson chi-square test. Then the data were correlated with histopathological diagnosis by multivariate analysis via logistic regression (LR) using IBM SPSS Statistics 23.0 (IBM Corp., Armonk, NY, USA). Comparison of groups was performed by univariate analysis, and significant variables were determined. Independent predictors that were selected out of these variables were analyzed by LR, and odds ratios (OR) were calculated. For the primary objective of the study, forward stepwise LR analysis was performed for each subgroup of predictors to test the effect of radiological predictors in diagnosis of AA. For the secondary objective, a new scoring system (NSS) was established according to the OR values of those independent variables. Reclassification method was used for comparing the performance of scoring systems. It was assumed that all patients would have been treated strictly according to the results of the scoring systems (PAS, ASS, and NSS). Patients with a score of 8 and higher for PAS, patients with a score of 7 and higher for ASS, and patients with a score of 12 and higher for NSS were assumed to be operated with prediagnosis of AA. The sensitivity, specificity, and receiver operating characteristic (ROC) curves were analyzed for the overall performances of PAS, ASS, and NSS. All tests were carried out using 0.05 as the significance level and the consistency among the scores was evaluated by Kappa test.

## 3. Results

A total of 1372 children were consulted with our department during the study period. Of these, 377 patients had pathologies other than AA and were excluded. Of the hospitalized patients with suspected AA (n=995), 37 cases had insufficient data and were also excluded (Figure 1). There were a total of 958 patients (437 girls and 521 boys) with a mean age of 10.8±4.2 years. Of these, 558 (58.2%) did not require surgical exploration. Of the remaining 400 patients who had undergone an appendectomy, 355 (88.8%) were histopathologically proven AA. Negative appendectomy rate was 11.3%. There was no missed appendicitis.

Group A (n=355) included patients with histopathologically proven AA whereas patients that were discharged without surgical exploration and patients with negative appendectomy constituted Group NA (n=603). Comparison of patient characteristics and findings between groups are summarized in Table 1. Male predominance was slightly higher in Group A (p=0.03). There was no difference concerning the mean age at operation between groups. Mean duration of symptoms and the rate of right lower quadrant tenderness were higher, and abdominal pain was more likely to be continuous in Group A whereas intermittent abdominal pain was more common in Group NA (p=0.001). Migration of pain, anorexia, bilious vomiting, pyrexia, guarding, rebound, positive heel drop test, gurgling, leukocytosis, neutrophilia, CRP elevation, scoliosis to the right side on X-ray, localized air-fluid level, localized gas deposition, appendicolith, increase in appendix diameter, and wall thickness and periappendiceal free fluid rates were significantly higher in Group A (p≤0.001).

Data of all 958 children in the study were used for the estimation of the regression coefficients and for the derivation of these results. LR analysis revealed 21 independent predictors with OR ranging from 1.667 to 30.195. Positive heel drop test was the most valuable independent predictor. Migration of pain, continuous abdominal pain and presence of an appendicolith on X-ray, guarding, rebound tenderness, thickened appendix wall, gurgling, neutrophilia, leucocytosis, increased appendix diameter on ultrasound, localized gas deposition on X-ray, bilious vomiting, periappendiceal free fluid, localized air-fluid level, scoliosis to the right side, pyrexia, right lower quadrant tenderness, increased CRP levels, anorexia, and male gender were the other predictors with decreasing OR values, consecutively. LR analysis of the predictors is depicted in Table 2.

To test the efficiency of radiological predictors, sensitivity and specificity were calculated using the data of all 958 children for three different groups of predictors, with only clinical and biochemical predictors, with only radiological predictors and with clinical, biochemical, and radiological predictors.

*Forward Stepwise LR Analysis—Evaluation without Radiological Predictors*. Seven predictors, positive heel drop test, continuous abdominal pain, migration of pain, duration of symptoms (>24 hours), bilious vomiting, guarding, and neutrophilia, were retained following multiple forward stepwise LR analysis of clinical and biochemical predictors. Sensitivity and specificity of this method for the diagnosis of AA were 84.6% and 94.8%, respectively (area under curve [AUC]=0.966, confidence interval [CI] =0.953-0.979, and p≤0.001).

*Forward Stepwise LR Analysis—Evaluation with Only Radiological Predictors*. Five predictors, appendix wall thickening and peri-appendiceal free fluid on the ultrasound, localized gas deposition, localized air-fluid level, and scoliosis to the right side on X-ray, were retained following multiple forward stepwise LR analysis of radiological predictors. Sensitivity and specificity of this method for the diagnosis of AA were 59.3% and 91.7%, respectively (AUC=0.836, CI=0.802-0.870, and p≤0.001).

*Forward Stepwise LR Analysis—Evaluation with Clinical, Biochemical, and Radiological Predictors*. Eleven predictors were retained following multiple forward, stepwise LR analysis of clinical, biochemical, and radiological predictors (positive heel drop test, continuous abdominal pain, migration of pain, gas deposition on X-ray, duration of symptoms [>24 hours], neutrophilia, guarding, free periappendiceal fluid on ultrasound, air-fluid level on X-ray, bilious vomiting, and fecalith on X-ray). Sensitivity and specificity of this method for the diagnosis of AA were 86.9% and 94.8%, respectively (AUC=0.978, CI=0.969-0.987, and p≤0.001).

**Figure 2:**
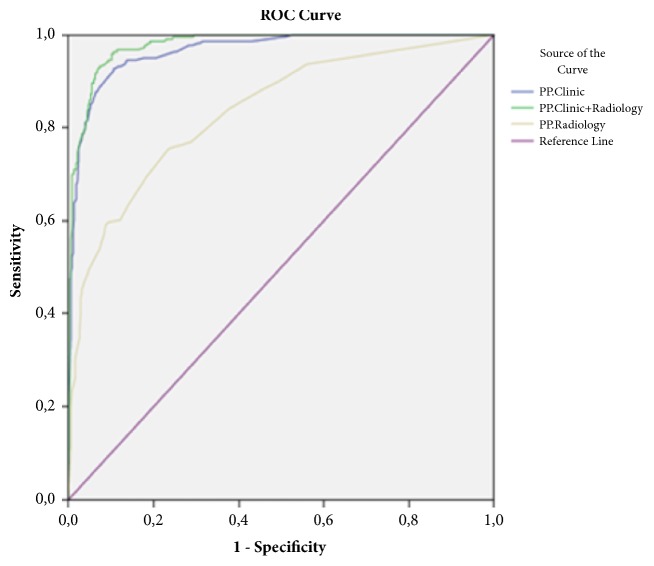
ROC curves of evaluation without radiological predictors (AUC=0.966, CI=0.953-0.979, and p≤0.001), with only radiological predictors (AUC=0.836, CI=0.802-0.870, and p≤0.001) and with clinical, biochemical, and radiological predictors (AUC=0.978, CI=0.969-0.987, and p≤0.001).

Figure 2 displays the ROC curves and Table 3 depicts the sensitivity and specificity rates of predictor subgroups.

*Establishment of NSS*. NSS scores were determined as 0.5 for predictors with OR:<3, 1 for predictors with OR:3-6, 2 for predictors with OR:6-9, and 3 for predictors with OR:>9. LR analysis of the predictors and NSS scores that were valued according to the OR's are summarized in Table 2. NSS score of 12 and higher was considered as the cut-off level for the diagnosis of AA.

**Figure 3:**
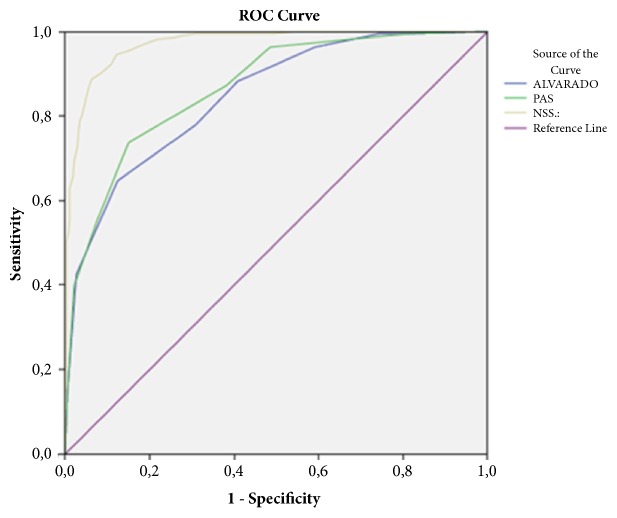
ROC curves for the overall performances of PAS, ASS, and NSS (AUC=0.868 CI=0.839-0.897, and p≤0.001) (AUC=0.847, CI=0.816-0.878, and p≤0.001) (AUC=0.972, CI=0.960-0.983, and p≤0.001), respectively.

For 958 patients, the sensitivity of ASS was 77.8%, the specificity was 70%, the PPV was 59.1%, and the NPV was 84.5%. The sensitivity of PAS was 55.2%, the specificity was 92.5%, the PPV was 80.8%, and the NPV was 78.3%. The sensitivity of NSS was 94.6%, the specificity was 87.9%, the PPV was 81.6%, and the NPV was 96.6% (Table 4). Kappa coefficient for NSS (0.797) was higher than of both ASS (0.441) and PAS (0.512) and indicated good agreement. The area under the ROC curve was 0.847 (95% CI=0.816–0.878) for ASS, 0.868 (95% CI=0.839-0.897) for PAS, and 0. 972 (95% CI=0.960-0.983) for NSS (Figure 3). As a result, these findings showed that NSS was significantly superior to ASS and PAS in diagnosing acute appendicitis.

For a better understanding of the performance of NSS regarding the duration of symptoms, true and false predicted values were classified according to different threshold durations (Table 5). NSS had an overall true negative rate of 55.8%, the false negative rate of 2.1%, true positive rate of 34.4%, and false positive rate of 7.7%. False positive rates were remarkably high in patients with duration of symptoms less than 24 hours.

## 4. Discussion

Diagnosis of AA is not always easy [1–4]. Reasons for this are variable symptoms and findings due to progressive inflammatory nature of the pathology, variations of appendix localizations, differences in pain thresholds, and unavailability of standard assessments of both clinicians and radiologists. Against all, the surgeon should combine all the accessible data while deciding for operation. In this study, the efficient predictors were identified, the efficacy of radiological data was assessed, and a new tool for a more accurate diagnosis of AA than existing scoring systems regarding sensitivity and specificity was developed.

A negative appendectomy rate up to 15-30% was regarded as acceptable a few decades ago [10–12]. Various scoring systems were introduced to diminish this rate to <10%. Popular ones were Alvarado and PAS [6, 7]. There were also other reports with different clinical scoring systems [10, 13, 14]. However, neither method was satisfactory to be the only method for decision-making. The most critical factor affecting the decision for surgery in suspected AA was the surgeon's experience and physical findings of repeated clinical examinations [8, 10, 15–19]. These methods were likely to be used for risk assessment and the criteria for surgical consultation [20]. In the study period, patients with suspected AA were evaluated and classified into three categories according to surgeon's preference; strong suspicion with positive physical examination findings who directly undergo surgical exploration, unclear ones with incompatible history and findings of physical examination or imaging studies who are observed with repeated physical examinations for at least 24 hours, and patients that are unlikely to be AA who are discharged. Only the hospitalized patients, either operated on or nonoperated on, may be evaluated and hence no comment can be made upon readmission (missed appendicitis) rate in this study. For the patients that have undergone surgery, the negative appendectomy rate was 11.3% which is acceptable and in parallel with the literature [10].

In previous studies, use of available imaging techniques alone was not sufficient for the diagnosis of AA but suggested superior results along with clinical judgment [2, 9, 10, 21–23]. This was also the case in our study. Radiological predictors alone achieved the sensitivity and specificity rates of 59.3% and 91.7%, respectively. A structured template for radiologists was proposed to minimize the bias in this user-dependent tool [24]. Computerized tomography (CT) was another tool to be used in the diagnosis of AA, especially in the USA but routine CT was found unnecessary, and the radiation hazard could only be minimized using PAS as an excluding tool and US as the primary imaging modality [5, 25, 26]. Although the place of radiography is controversial in the diagnosis of AA, our results show that it is useful together with ultrasonography [1, 26].

Laparoscopy has been used for both diagnosis and treatment of AA, and its availability and ease of use may increase unnecessary surgical explorations regarding negative appendectomy rates [27, 28]. Although laparoscopy was not performed in all patients, its ease of use might have contributed to our negative appendectomy rate by being a facilitating factor in decision-making for surgery. In a previous study, the percentage of removing normal appendix was the highest in laparoscopy (9%) versus open (3%) and observation (3%) [28].

The surplus number of scoring systems is an indicator that the ideal system has not yet been found [3, 4, 29–31]. In a systematic review for the performance of ASS in predicting appendicitis, authors stated that the heterogeneity was apparent between studies where they have found a nonsignificant trend towards overprediction in the low risk strata and a significant overprediction in the intermediate risk category and high risk strata in children [32]. In our study, ASS with a cut-off level of 7 had sensitivity and specificity rates of 77.8% and 70%, respectively. Its PPV rate was the worst among scoring systems (59.1%) but had a moderate success rate of NPV (84.5%). What is more, Kappa coefficient was the lowest among the scoring systems that suggests near-fair agreement (Table 4). Likewise, PAS was also found to have very heterogeneous results for most cut-off points in a recent meta-analysis, although the authors have grouped studies with similar inclusion criteria [33]. They stated that the heterogeneity was probably due to the examiner-dependent variables of PAS and concluded as the imaging studies should be added to history taking, physical examination, laboratory test, and PAS in patients with suspected AA. In our study, PAS had the lowest sensitivity (55.2%) and highest specificity (92.5%) rates. Its PPV rate (80.8%) was close to NSS, higher than ASS with a Kappa coefficient of 0.512 that suggests moderate agreement. On the other hand, NSS had the highest rate of sensitivity (94.6%), a high rate of specificity (second after PAS), the highest rates of PPV (81.6%), and NPV (96.6%) with a Kappa coefficient of 0.797 that suggested a good agreement (Table 4).

Although our scoring system achieved relatively superior results, NPV of AA was evidently high (10%) in patients whose symptom duration was less than 24 hours (Table 5). This points out that the ideal clinical scoring system should be established and adopted according to the pathological course of the disease. In a perfect scoring system, the predictors should be well determined according to the course of the inflammation, and it should discriminate between acute, exudative, and complicated phases. This study represents as a preliminary one for determining all the accessible significant variables and setting a new scoring system that would be tested prospectively. Artificial neural network studies may also be preferred for comparison on this subject.

Detailed history taking and recording of the objective findings on prestructured forms was previously shown to be useful, especially in referral centers with high patient and clinician volume [34]. In accordance with this statement, all initial assessments of the patients were made on standardized prestructured evaluations forms in a prospective study fashion. However our study has also some limitations due to its retrospective nature. The reclassification method that we have used may have produced optimistic results that need to be tested prospectively. Alternative methods could not be performed due to the limited number of patients in the subgroups. Clinical observation was previously shown to improve the ability to diagnose AA but the records of the duration of clinical observation for each patient could not be reached [35]. Operation notes were also not standardized regarding the localization of the appendix; hence, no comments may be made upon this factor in our study. The timing of the diagnostic tests, objectivity, and experience of radiologists are also other varying factors that may have interfered with our results.

## 5. Conclusion

Although limited, radiography and ultrasound improve the sensitivity rate in patients with suspected AA. With reclassification method, NSS performs superiorly to PAS and ASS, regarding sensitivity and specificity without any age limitation. Scoring systems can be of assistance in setting the diagnosis of acute appendicitis, but none have the adequate predictive values in assessing acute appendicitis and none can be used as an exclusive standard in setting the diagnosis of acute appendicitis in children, yet. Pathophysiological phase-intended variables may improve the success rate of scoring systems.

## Figures and Tables

**Table 1:** Comparison of patient characteristics and findings between study groups (GI: gastrointestinal, CRP: C-reactive protein).

	**Group Non-appendicitis**	**Group Appendicitis**	p
n	603	355	
Gender distribution (Female/Male)	45.8% / 54.2%	39.7% / 60.3%	**0.03**
Mean age (years)	10.6±4.6	10.9±4.1	>0.05
Mean duration of symptoms (hours)	30.51±30.60	39.84±35.63	**0.001**
Continuous abdominal pain (%)	20.2	79.2	**0.001**
Intermittent abdominal pain (%)	79.6	20.8	**0.001**
Migration of abdominal pain (%)	10.6	64.7	**≤0.001**
Nausea (%)	10.1	5.9	**≤0.001**
Anorexia (%)	54.5	77.3	**≤0.001**
Bilious vomiting (%)	12.1	46.6	**≤0.001**
Pyrexia (%)	11.1	33.9	**≤0.001**
Right lower quadrant tenderness (%)	95.1	100	**0.001**
Guarding (%)	46	88.2	**≤0.001**
Rebound (%)	23	70.1	**≤0.001**
Positive heel drop test (%)	5.9	65.6	**≤0.001**
GI motility changes (%)	7.2	10.4	>0.05
Gurgling (%)	4.9	26.2	**≤0.001**
Leukocytosis (%)	55.3	87.8	**≤0.001**
Neutrophilia (%)	44.4	83.3	**≤0.001**
CRP elevation (%)	33.3	65.6	**≤0.001**
Negative urinalysis (%)	79.6	85.9	0.05
Scoliosis to right side (%)	19.4	51.6	**≤0.001**
Localized air-fluid level (%)	21.4	56.6	**≤0.001**
Localized gas deposition (%)	15.8	51.6	**≤0.001**
Appendicolith (%)	0.5	5.9	**≤0.001**
Appendix diameter>7mm (%)	11.1	41.6	**≤0.001**
Appendix wall thickening (%)	11.9	49.3	**≤0.001**
Periappendiceal free fluid (%)	18.6	52.5	**≤0.001**

**Table 2:** Results of logistic regression and determination of new scoring system (NSS) scores according to odds ratios (CI: confidence interval, NSS: new scoring system, and CRP: C-reactive protein).

**Predictor**	**Odds ratio**	**95**%** CI**	**NSS** **Score**
Male gender	1.667	1.188 – 2.339	0.5
Continuous abdominal pain	15.022	9.981 – 22.611	3
Migration of pain	15.637	10.187 – 24.002	3
Anorexia	2.853	1.964 – 4.143	0.5
Bilious vomiting	5.285	3.383 – 8.256	1
Pyrexia	4.110	2.695 – 6.267	1
Right lower quadrant tenderness	4.090	2.121 – 7.882	1
Guarding	8.806	5.585 – 13.886	2
Rebound tenderness	7.863	5.416 – 11.417	2
Positive heel drop test	30.195	18.230 – 50.011	3
Gurgling	6.892	3.977 – 11.944	2
Leucocytosis	5.809	3.705 – 9.107	2
Neutrophilia	6.216	4.142 – 9.3308	2
Increased CRP	3.816	2.692 – 5.409	1
Scoliosis to the right side	4.432	3.077 – 6.384	1
Localized air-fluid level	4.769	3.327 – 6.836	1
Localized gas deposition	5.694	3.894 – 8.326	1
Appendicolith	12.031	2.689 – 53.825	3
Increased appendix diameter	5.705	3.768 – 8.638	1
Thickened appendix wall	7.214	4.810 – 10.821	2
Periappendiceal free fluid	4.792	3.316 – 6.924	1

**Table 3:** Sensitivity and specificity rates for clinical, biochemical, radiological, and combined predictors.

**Predictor sub-groups**	**Sensitivity**	**Specificity**
Clinical and biochemical predictors	84.6%	94.8%
Radiological predictors	59.3%	91.7%
Clinical, biochemical, and radiological predictors	86.9%	94.8%

**Table 4:** Diagnostic performance of ASS, PAS, and NSS (PPV: positive predictive value, NPV: negative predictive value, ASS: Alvarado Scoring System, PAS: Pediatric Appendicitis Score, and NSS: New Scoring System).

		**ASS** **(Cut-off: 7)**	**PAS** **(Cut-off: 8)**	**NSS** **(Cut-off: 12)**
**# of patients**	True positive	271 (28.3%)	192 (20%)	336 (35.1%)
	False positive	188 (19.6%)	46 (4.8%)	101 (10.5%)
	False negative	77 (8%)	156 (16.3%)	12 (1.3%)
	True negative	422 (44.1%)	564 (58.9%)	509 (53.1%)

**Sensitivity (**%**)**		77.8	55.2	**94.6**
**Specificity (**%**)**		70	92.5	**87.9**
**PPV (**%**)**		59.1	80.8	**81.6**
**NPV (**%**)**		84.5	78.3	**96.6**
**Kappa coefficient**		0.441	0.512	**0.797**

**Table 5:** Relation between duration of symptoms and accuracy of the New Scoring System (NSS).

**Duration of symptoms**	**True negative**	**False positive**	**False negative**	**True positive**
**=<12 hours (n=402)**	63.2%	**10.2**%	2.5%	24.1%
**12-24 hours (n=214)**	49.1%	**10.7**%	0.5%	39.7%
**24-48 hours (n=184)**	52.2%	2.7%	2.7%	42.4%
**>48 hours (n=158)**	50.6%	3.2%	1.9%	44.3%
**Total**	55.8%	7.7%	2.1%	34.4%

## Data Availability

The data used to support the findings of this study are available from the corresponding author upon request.
